# Sisymbrium Officinale (the Singers’ Plant) as an Ingredient: Analysis of Somatosensory Active Volatile Isothiocyanates in Model Food and Drinks

**DOI:** 10.3390/foods10020308

**Published:** 2021-02-03

**Authors:** Patrizia De Nisi, Gigliola Borgonovo, Samuele Tramontana, Silvia Grassi, Claudia Picozzi, Leonardo Scaglioni, Stefania Mazzini, Nicola Mangieri, Angela Bassoli

**Affiliations:** 1 Gruppo Ricicla, Department of Agricultural and Environmental Sciences-DISAA, University of Milan, Via Celoria 2, I-20133 Milano, Italy; patrizia.denisi@unimi.it; 2 Department of Food, Environment and Nutrition-DeFENS, University of Milan, Via Celoria 2, I-20133 Milano, Italy; gigliola.borgonovo@unimi.it (G.B.); tecnologo.tramontana@gmail.com (S.T.); silvia.grassi@unimi.it (S.G.); claudia.picozzi@unimi.it (C.P.); leonardo.scaglioni@unimi.it (L.S.); stefania.mazzini@unimi.it (S.M.); nicola.mangieri@unimi.it (N.M.)

**Keywords:** *Sisymbrium officinale*, isothiocyanates, HS-SPME-GC/MS, TRPA1 ion channel, somatosensory, trigeminal perception

## Abstract

*Sisymbrium officinale* (L.) Scop. (hedge mustard) is a wild common plant of the Brassicaceae family. It is known as “the singers’ plant” for its traditional use in treating aphonia and vocal disability. The plant is rich in glucosinolates and isothiocyanates; the latter has been demonstrated to be a strong agonist in vitro of the Transient Receptor Potential Ankirine 1 (TRPA1) channel, which is involved in the somatosensory perception of pungency as well as in the nociception pathway of inflammatory pain. Volatile ITCs are released by the enzymatic or chemical hydrolysis of GLSs (glucosinolates) during sample crushing and/or by the mastication of fresh plant tissues when the plant is used as an ingredient. Some functional food and drink model preparations have been realised: honey enriched with seeds and flowers, infusions, cold drink (voice drink), artisanal beer, and a fermented tea (kombucha). Using SPME-GCMS chromatography, we analysed samples of the plant and of the food preparations adopting conditions that simulate the release of isothiocyanates (ITCs) during oral assumption. Two active compounds, iso-propylisothiocyanate and 2-butylisothiocyanate, have been assayed. The concentration of ITCs varies according to temperature, pH, grinding conditions, and different plant organs used. Kombucha-type fermentation seems to eliminate the ITCs, whereas they are retained in beer. The ITCs’ concentration is higher when entire seeds and flowers are used.

## 1. Introduction

*Sisymbrium officinale* (L.) Scop. (Brassicaceae) (SO) is an annual plant that is spread mostly in the Eurasiatic Region and North Africa. The plant is also commonly named “hedge mustard”, “erysimum”, or “the singers’ plant”, and it is known for its traditional use in the treatment of mild voice and respiratory discomfort. SO properties and uses has been recently reviewed [1].

The chemical markers [2] of SO are glucosinolates (GLSs) and isothiocyanates (ITCs), which are their main metabolic breakdown products, and these are also commonly found in many Brassicaceae. Historically, sulphured compounds are reported to stimulate mucosal secretion and expectoration from the upper respiratory tract. [3]. The pharmacology of SO shows anti-inflammatory, analgesic, antitussive, myorelaxant [4], a broad spectrum of antimicrobial [5] activity and also antimutagenic properties [6]. The effect of SO phytopreparates on alleviating symptoms in patients with various degrees of vocal trait disability has been recently reported [7].

As in other plants of the Brassicaceae family, GLSs are present in SO with their corresponding ITCs, which are formed by enzymatic processes mediated by myrosinase and/or by chemical hydrolysis that could occur during the plant manipulation, storage, and processing. The relative content of GLSs and ITCs is often difficult to establish due to the transformations of the first into the latter in the same matrix. Moreover, the two classes of compounds have very different chemical features: polar GLSs are generally analysed by UV spectroscopy and/or HPLC-DAD, whereas gas chromatography–mass spectrometry (GCMS) is more suitable for volatile ITCs.

**Figure 1:**
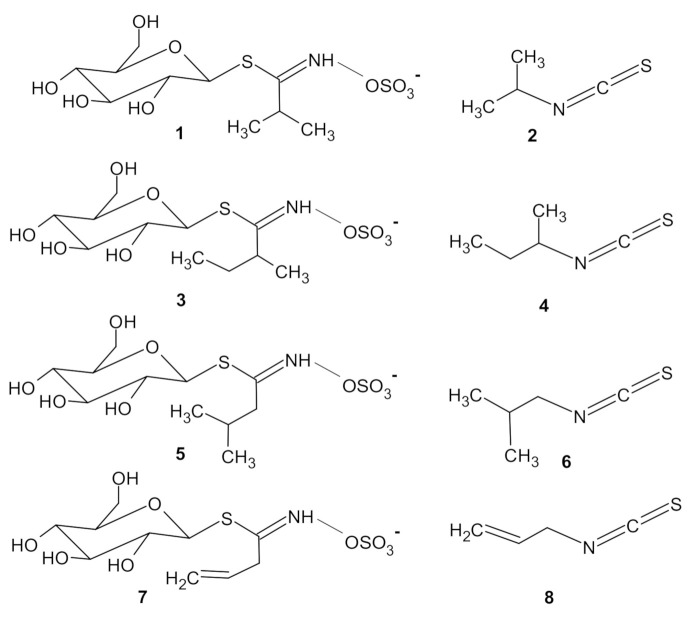
Glucosinolates (left) and isothiocyanates (right) previously identified in *Sisymbrium officinale*. **1**: glucoputranjivin; **2**: iso-propylisothiocyanate—IPITC; **3**: glucocochlearin; **4**: sec-butylisothiocyanate—SBITC; **5**: glucojiabutin; **6**: iso-butylisothiocyanate—IBITC; **7**: sinigrin; **8**: allyl-isothiocyanate—AITC.

The literature [8] identifies glucoputranjivin **1** (Figure 1) as the main glucosinolate in SO; its corresponding isothiocyanate is iso-propylisothiocyanate (IPITC, compound **2**)**.** Other GLSs as glucocochlearin **3**, glucojiabutin **5**, and sinigrin **7** are also present together with their congener ITCs, compounds **4** (sec-butylisothiocyanate, SBITC), **6** (iso-butylisothiocyanate, IBITC) and **8** (allyl-isothiocyanate, AITC) [5,9,10]. Some of these compounds were previously identified in other plants as cochlearia [11] or rocket [12].

Compounds **1**–**8** are shown in Figure 1. 

A comparative analysis of GLSs in wild and cultivated SO has been recently published [13]. An extensive analysis of volatile compounds obtained by the hydrodistillation by GCMS was described and showed the presence of at least 42 different volatile compounds in SO, including several ITCs. Among volatiles, these compounds seem to be the most active from a biological point of view [5].

SO is commonly regarded as a medicinal plant, but some sporadic gastronomic uses are also reported; the plant is in fact a wild mustard, with a mild bitter and pungent taste profile, and it can be used likewise for food preparations. Recently, several food and drink preparations with SO have been described, including a “voice drink”, a cold drink with SO, and other active ingredients for voice care [14]. 

SO and many other plants are recorded in the “Herbs for Voice Database”, which is an open database of plants used for voice discomfort and airways pathologies [15]. The analysis of this database shows that the Transient Receptor Potential Ankyrin 1 (TRPA1) channel, which is involved in the somatosensory perception of pungency as well as in the nociception pathway of inflammatory pain, is the most represented molecular target for bioactive compounds in these plants. Compounds **2** and **4** from SO are in fact potent agonists in vitro of the TRPA1 channel [16]. Therefore, the analysis of these ITCs in SO become relevant to determine the fraction of volatiles that can contribute to the overall bioactivity reaching the nose and upper airways by inhalation and retronasal olfaction, as normally occurs drinking herbal teas or during the mastication of tablets. 

The aim of this work is to develop new food and drink products with SO; to do that, we need an efficient method to quantify the presence of volatile ITCs in SO plant and in phytopreparates therefrom, as well as in food and drink preparations. The amount of free isothiocyanates in fact depends not only from the initial content of glucosinolates but also on the type of processing adopted. Temperature, pH, and all factors affecting the enzymatic or chemical hydrolysis of GLSs to ITCs may change the concentration of ITCs in the final product [17]. 

In the present work, we describe the preparation of model food and drinks with SO seeds, flowers, and leaves as functional ingredients. Released ITCs are analysed by headspace solid-phase microextraction (HS-SPME) coupled with gas chromatography-mass spectrometric (GC-MS). The concentration of released ITCs varies according to temperature and grinding conditions. Kombucha-type fermentation seems to eliminate the ITCs, whereas they are abundant in beer. In three samples, the concentration of released ITCs is higher than that of the reference sample, which is a commercial tablet used in the treatment of aphonia.

## 2. Materials and Methods 

### 2.1. Plant Material

*Sisymbrium officinale* was cultivated at the Faculty of Food and Agricultural Sciences at the University of Milano [18]. For each food and drink, preparations different organs of SO plant were used. Plants have been harvested during the flowering season (May–July 2017–2018–2019) and seeds collected in August, September, and October 2017, 2018, and 2019. The aerial parts (leaves, flowers) of the plant were dried at room temperature and stored in paper bags. Seeds were stored in vacuum at 4 °C. In some preparations (honey, voice drink, kombucha), fresh samples were harvested from living plants and used immediately.

### 2.2. Food and Drink Preparations

#### 2.2.1. Honey Enriched with SO Seeds or Flowers

For the preparation, we used commercial acacia honey, which is fluid, clear, and does not crystallises during storage. Ten glass vessels were sterilised by boiling for 15 min, filled with honey (15 g) and SO seeds (0.6 g) or fresh flowers (0.4 g), mixed for 60 s, sealed, and stored at room temperature in a cabinet for 4 weeks before analysis. Samples with plain honey were stored in the same conditions and used as control. For CG/MS analysis, samples of honey (0.5 g) were dissolved in deionised water (2 mL) and analysed; two samples (S3, S5) were grinded for 60 seconds in a mortar before analysis. 

#### 2.2.2. Infusion

An infusion was prepared using dry commercial SO (whole aerial parts of plant, Erboristeria Ape Regina, Milano, Italy) and following the conventional dosage suggested for herboristic use. Tap water (1 L) was heated to the boiling point; then, heating is stopped, after which SO (13 g) was added and allowed to infuse for 10 min in a closed vessel. 

#### 2.2.3. Voice Drink 

A model voice drink was prepared using fresh seeds and flowers from our cultivation. Tap water (1 L) was heated to the boiling point; then, SO seeds (0.7 g) and flowers (0.25 g) were added, and the drink was allowed to infuse for 10 min in a closed vessel. For GC/MS analysis, flowers (25 mg) and seeds (70 mg) are added to deionised water (1 mL) in a test head space vials. 

#### 2.2.4. Beer (the “Singers’ Beer”)

Artisanal beer was prepared following a standard fermentation process [14]. Malt (Sanapils, Mr.Malt, 70 g) is added to 6 L of water at 60 °C, allowed to infuse for 20 min, and then filtered. Malt extract (Pilsner baumann, Weiermann, 650 g) is added and the solution is boiled; hop (fuggle cones AA595, 15 g in three portions) and SO (6 g) (commercial dry SO aerial parts, Erboristeria Ape Regina, Milano) are added in an infusion bag; after 5 min, the bag is removed, and the solution is allowed to cool to 20 °C and inoculated with yeast (Safale-S-04, Fermentis, 5.5 g). Fermentation is kept at 20 °C for 7 days; then, sugar (5 g/L) is added, and beer is bottled and stocked at room temperature for 2 weeks and then in a room at 10 °C. For GC/MS analysis, samples of 2 mL were used. In these samples, the abundant presence of CO_2_ is required to raise the delay time of mass spectrometer to 1 min. 

#### 2.2.5. Kombucha 

A fermented drink in the kombucha style (“Erbucha”) was prepared. One litre of distilled water was brought to boil and then added with 5 g of SO flowers and 40 g of sucrose (CAS 57-50-1; Sigma-Aldrich, Milan, Italy). The solution was left to infuse for 20 min and after cooling was filtered with filter paper, transferred into sterile glass vessel (1 L), and the Symbiotic Colony of Bacteria and Yeast (SCOBY) was added. The jar was covered with a sterile gauze and left to ferment at room temperature for 12 days. The SCOBY aliquots (5 mL) for GCMS analysis were taken and stored at −20 °C every three days. Before the analysis, samples were allowed to reach room temperature and immediately analysed. 

#### 2.2.6. Tablet

Commercial tablets for aphonia (Voxyl, Pierre Fabre) were grinded; the powder (500 mg) was dissolved in deionised water (2 mL) and analysed. Tablets contain 15 mg of dry extract of SO (*summitas cum floribus*). 

### 2.3. Microbiological Analyses 

In order to evaluate the microbiological quality of the different plant material, approximately 10 g of seeds, leaves, and flowers were diluted in sterile peptone water (10 g/L bacto peptone, pH 6.8) and homogenised in Stomacher 400 Circulator (Seward, Worthing, UK) for 5 min. After appropriate dilutions, samples were plated in different media in order to evaluate their initial microbial contamination. Total bacterial count (TBC) was determined after incubation of tryptic soy agar (TSA, Scharlab, Barcelona, Spain) plates at 30 °C for 48–72 h. Yeasts and moulds were plated in Yeast Glucose Chloramphenicol Agar (YGC, Scharlab) and incubated at 25 °C for 3–5 days. *Escherichia coli* and *Bacillus cereus* counts were estimated after incubation at 44 °C for 24–48 h in Tryptone Bile X-Glucuronide (TBX) agar (Merck KGaA, Darmstadt, Germany) and at 37 °C for 48 h in *Bacillus cereus* selective Agar (PEMBA) (Merck) respectively. For beer samples, 10 mL were taken and analysed before bottling, after one day and after 30 days. Yeasts were determined after incubation in YGC at 25 °C for 3–5 days and lactic acid bacteria after incubation in MRS—de Man Rogosa Sharp (BD, Oxford, UK) at 37 °C for 3 days. Beer without the addition of SO was used as negative control.

Domestic Kombucha was used for the fermentation, which contained two yeast strains (*Zygosaccharomyces kombuchaensis* and *Dekkera bruxellensis*) and one acetic acid bacteria (*Komagataeibacter rhaeticus*). Microbial composition was analysed after the fermentative process to evaluate if the change in the substrate (SO instead of green or black tea leaves) could change the ratio and the species of microorganisms. In brief, serial dilutions were prepared starting from 10 mL of liquid and 10 g of SCOBY homogenised in Ringer’s solution (Oxoid, Milan, Italy) through a stomacher (Stomacher400 circulator; Seward Medical, London, UK). Dilutions were plated on Nutrient Agar added with 100 μg/mL of cycloheximide at 30 °C for 48–72 h and on YGC at 25 °C for 3–5 days for bacteria and yeast enumeration, respectively. Colonies from the highest dilutions were isolated and subjected to DNA extraction following the protocol of Sambrook and Russel [19] for bacteria and of Vigentini et al. [20] for yeasts. Then, the extracted DNAs were subjected to amplification of 16s rRNA gene for bacteria [21] and of the 26S rDNA D1/D2 domain [22] and then identified after been sequenced by an external provider (Eurofins Genomics, Ebersberg, Germany).

### 2.4. HS-SPME Coupled with GCMS Analysis 

Iso-propylisothiocyanate (CAS 2253-73-8), sec-butylisothiocyanate (CAS 4426-79-3), methanol are from Sigma-Aldrich (Milan, Italy). The determination of ITCs was performed following literature methods [23,24,25]. Volatiles analyses were conducted with GC 7890A gas chromatograph associated to 5975C mass spectrometer (Agilent, Palo Alto, CA, USA).

For the analysis of ITCs in food preparations, two calibration curves have been built, using IPITC **2** (80% methanol in water solution) as a standard at the concentrations of 0.25–1 ppm and 5–40 ppm respectively (Figure S1). 

Autolysed samples were prepared by dissolving samples (as such, in case of liquids or dissolved in water, in case of solids) in a GC headspace vial (Agilent Technologies), which was rapidly sealed with a magnetic screw cap equipped with silicon/polytetrafluoroethylene septa (PTFE). Headspace (HS) volatile compounds were collected using a SPME-Solid Phase MicroExtraction fiber (Supelco, Bellefonte, PA, USA) 2 cm × 50/30 µm thickness coated with DVB/CAR/PDMS (divinylbenzene/carboxen/polydimethylsiloxane). Initially, vials were tempered at 37 °C for 2 min. Then, the volatiles were extracted by exposing the fiber to the vial headspace for 5 or 10 min under continuous agitation and heating at different temperature (37 or 80 °C) according to the preparations. The extracted volatiles were desorbed in the GC injection port for 2 min at 230 °C in splitless mode. Incubation of the vials, extraction, and desorption of the volatiles were performed automatically by a CombiPAL autosampler (CTC Analytics, Zwingen, Switzerland). Chromatography was performed on a DB-5 MS (30 m × 0.25 mm × 0.25 μm) column (J&W Scientific, Folsom, CA, USA) with helium as carrier gas at a constant flow of 1.0 mL/min. GC interface, MS source, and quad temperatures were 260, 230, and 150 °C, respectively. Oven temperature conditions were 40 °C for 2 min, then 15 °C/min ramp until 240 °C and held at for 4 min. Mass spectra were recorded in scan mode in the 30 to 300 mass-to-charge ratio range by a 5975B mass spectrometer (Agilent Technologies, Santa Clara, CA, USA) at an ionisation energy of 70 eV and a scanning speed of 7 scans/s. Chromatograms and spectra were recorded and processed using the Enhanced ChemStation software, version MSD Chemstation E. 0200.493 (Agilent Technologies, Santa Clara, CA, USA). Details of operative conditions for GC and autosampling and mass spectra of IPITC are available as Supplementary material Figures S2–S4.

## 3. Results 

### 3.1. Identification of ITCs

**Figure 2:**
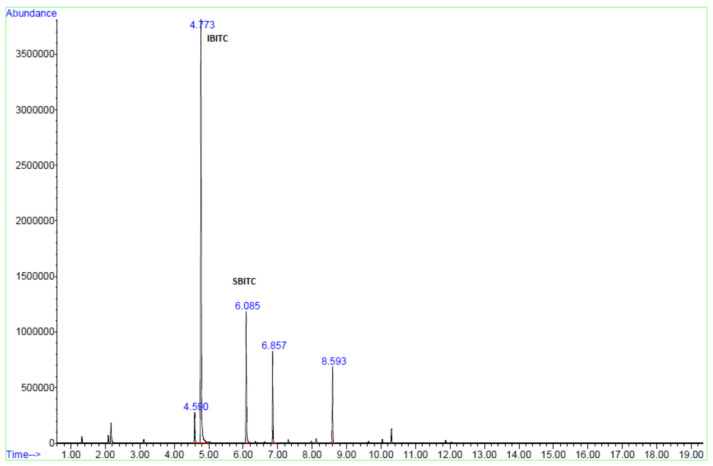
Total ion chromatogram (TIC) of volatile from headspace solid-phase microextraction (HS-SPME)-gas chromatography–mass spectrometry (GC/MS) analysis *Sisymbrium officinale* (L.) Scop. (Brassicaceae) (SO) flowers. First, 25 mg of SO flowers were weighted directly in a vial with 1 mL of distillated water, chopped, and quickly closed; exposure time of SPME fiber and temperature: 10 min at 37 °C.

The identification of released ITCs in samples was done using reference compounds **2** and **4** and by comparison of mass spectra with those in NIST 08 database. As an example, the total ion chromatogram (TIC) spectrum of volatile ITCs released from SO flowers in water is reported in Figure 2, showing two main peaks of interest, one at a retention time 4.773 min and another at a retention time 6.085 min. 

Peaks at 4.590, 6.857, and 8.593 are spurious peaks identified as silyl derivatives from the injection system (Figure S5). The mass spectrum of the main volatile compound at 4.773 min is identical to those reported for IPITC in the NIST—National Institute of Standards and Technology library and in Blazevic et al. [5]; therefore, the main volatile compound can be identified as compound **2**. 

For the peak at retention time 6.085 min, the database indicated two possible structures of the isothiocyanates with iso-butyl or sec-butyl side chain. The latter derives from glucocochlearin **3**, which was previously identified in our samples; a direct comparison with an authentic sample allowed identifying this peak as SBITC, compound **4**. No other ITCs could be identified in our samples; therefore, for the following experiments the total ITCs content was calculated by the sum of compounds **2** and **4**. 

### 3.2. Effect of Temperature, Time, and Grinding on ITCs Release

Before analysing food samples, we studied the effects of three parameters on the release of volatile ITCs from fresh SO flowered sprouts: temperature and time of treatment, and grinding of plant material (sample S1). Tests are conducted in a vial for HS- SPME by weighting 25 mg of flowers and 1 mL of water. Vials were filled and rapidly closed. The three parameters were varied to mimic, at least in part, the ordinary conditions of assumption of food and drink, including mastication. In these experiments, the total ITCs content was calculated as the sum of compounds **2** and **4** obtained by the calibration curves. Results of preliminary experiments are reported in Table 1. Additional data are reported in Supplementary material, Table S1.

S1/1 contains SO flowers that were heated for 10 min in water at 37 °C without grinding, whereas S1/2 is the same sample rapidly grinded in the vial before SPE analysis, thus simulating the mastication in the mouth. The comparison shows that grinding increases remarkably the release of ITCs in the sample; this can be attributed to the release of myrosinase from tissues, which favours the enzymatic hydrolysis of glucosinolates to volatile isothiocyanates. A similar effect is common in other food preparations; for instance, during the mastication of rockets leaves or by grinding mustard seeds in mustard under the teeth, the release of pungent AITC is immediately perceived in oral cavity and nose. 

The effect of temperature is shown comparing entries S1/2 and S1/3, which is heated at the temperature of 37 °C and 80 °C, respectively. In this case, a marked decrease of total ITCs content (from 0.8 to 0.12) is observed; this could be due to the inactivation of myrosinase in the plant material at this temperature [26]. Changing time from 5 to 10 min did not affect the release of ITCs. 

### 3.3. Food Samples, Choice, and Preparation

Several samples of model food and drink preparations with SO were prepared. The effects of bioactive volatiles ITCs are thought to occur mainly locally in the upper airways and oropharyngeal cavity, i.e., in the nose, mouth, throat, larynx, and vocal cords [7]. Here, the pungency of ITCs, due to the interaction with TRPA1 ion channel, is perceived. For food preparations, we focussed on those expected to maximise the release of ITCs during/after the ingestion and possibly having a good taste at the same time. 

The first sample we prepared is honey added with fresh erysimum flowers or seeds and allowed to stand for 4 weeks. 

Honey is often used as an emollient for throat pain and mild affections of respiratory tract; honey with SO flowers (samples S2 and S3) or seeds (samples S4 and S5) can be stored for a long time at room temperature, preserving plant parts from spoiling; it can be consumed as such in a spoon or dissolved in hot water to make a sweet hot drink. The effect on the release of ITCS by leaving whole material or by grinding seeds or flowers during the heating, in order to simulate the mastication, was also studied. 

An infusion (S6) was prepared with commercial dry plant (whole aerial parts) in infusion in hot water, as suggested for standard herbal tea. As an alternative, a model voice drink (S7) was prepared with fresh seeds and flowers in water. 

Two samples were prepared to explore the effects of fermentation on SO active ITCs. Artisanal beer (S8) (“the singer’s beer”) was prepared by adding commercial dry SO in infusion during the malt fermentation process. A fermented tea with SO (S9) was prepared. Fermented drinks in the Kombucha style, also known as “bubble teas”, are becoming popular among consumers for their pleasant sour taste, light carbonation, low alcohol content, and benefits on health [27]. 

As a reference for commercial products using erysimum as an herbal remedy for voice, we analysed in the same experimental conditions a commercial tablet with SO extracts commonly used for aphonia (S10). All food and drink samples are listed in Table 2.

### 3.4. Analysis of Samples

Microbiological analyses of seeds, leaves, and flowers revealed a good hygienic quality of all the parts of SO plants since all the results presented values that are below the limit proposed by European Commission and International Commission on Microbiological Specifications for Foods [28] (Table 3).

Model samples for the GC/MS analysis were prepared in order to mimic as far as possible the real food and drink preparation and consumption modalities.

To minimise the loss of volatiles, autolysed samples were prepared by dissolving samples in water in a vial, which was rapidly sealed with a magnetic screw cap equipped with silicon/polytetrafluoroethylene septa (PTFE). The two main pungent ITCs, compounds **2** and **4**, were determined by triplicate analysis; the average value is reported in Table 2. Since other ITCs could be not determined in these experimental conditions, the total ITCs content was calculated as the sum of compounds **2** and **4**. Then, the total ITCs release has been calculated in a standard portion of each food or drink as in its common use, which is assumed to be 1 spoon for honey, 1 teacup = 125 mL for infuse and voice drink, 1 can = 330 mL for beer and 1 tablet.

Samples of honey showed a low ITCs release; only sample S5, containing seeds and submitted to grinding before analysis, has an ITCs content much higher than the others, confirming that ITCs are well preserved in the plant material and their release is favoured by the activation of enzymatic hydrolysis. Since the precursor glucosinolates are most abundant in flowers than in seeds [13], the lower content of ITCs in honey with flowers can be related to a loss due to various enzymatic processes, which can involve flowers more than seeds. In fact, flowers’ tissues have about 90% water, whereas seeds are mainly constituted by oil and are therefore protected by enzymatic hydrolysis until grinding occurs. 

Samples of honey have an ITCs release comparable or higher to that of the commercial tablets (S10).

The analysis of infusion (S6) showed a release of active ITCs of 38.56 µg/kg for compound **2** and 8.29 µg/kg for compound **4**. 

“Classic“ infusion with whole dried plant has a total ITCs content per portion higher than that of the voice drink (5.85 µg/portion vs. 0.95 µg/portion), indicating that the contribution of ITC released from dried leaves is relevant. On the other hand, the commercial infusion is made by the whole dried plant, including those parts containing low or no active principles, and it has an overall herbaceous, mild bitter, and astringent flavour and taste. Instead, the voice drink is made with seeds and flowers and results cleaner with a mild taste profile where the pungency of ITCs can be easily perceived. 

The peculiar taste and somatosensory notes of ITCs are not easily detected in the fermented drinks, where they are covered by a number of taste active compounds and volatile flavours. 

The ITCs content resulted quite high in beer (S8), with a value of 7.62 µg/portion considering a can as a standard portion (330 mL); calculating the ITC concentration in the same volume, the content is about half that of infusion (17.84 vs. 31.03 µg/L). 

Whereas the “singers’ beer” could be a new and attractive product for consumers, it is clear that the presence of alcohol could not be recommended in a functional food or drink preparation. 

The presence of *Sisymbrium officinale* did not affect fermentation with *Saccharomyces cerevisiae* in the preparation of beer (S8); actually, yeast counts before bottling and after one day were approximately around 10^10^ CFU/mL both for S8 samples and for the negative control. After one month, the counts decreased to approximately 10^4^ CFU/mL, and no microbial contaminants were found in both samples at any time of analysis. 

As concerns the kombucha-type beverage (S9), the obtained drink has a pleasant, sour taste profile, but the sample analysis revealed that only traces of compound **2** can be detected in samples after one day of fermentation, whereas after three days of fermentation, it is possible to observe the formation of a very complex mixture of volatile compounds where isothiocyanates **2** and **4** cannot be detected. This can be explained with the fact that the fermentation process produces organic acids that are lowering pH to values below 3.6; Gil and MacLeod [30] reported that isothiocyanate is the main derivative that can be liberated from sinigrin at pH between 4 and 5, while with pH below 3.7, the main compound is cyanate. Furthermore, molecular analysis of sample S9 after 12 days of fermentation showed a change in microbial composition. The predominant yeast remains *Dekkera bruxellensis*, while we did not find any more *Zygosaccharomyces kombuchaensis.* As concerns acetic acid bacteria, we were able to identify different species of *Komagataeibacter* (Komagataeibacter hansenii and *Komagataeibacter saccharivorans*) together with *Acetobacter musti*. It has already been demonstrated by different authors that the microbial consortium of the SCOBY is not stable, and predominant species may vary with fermentations [31,32]. 

**Figure 3:**
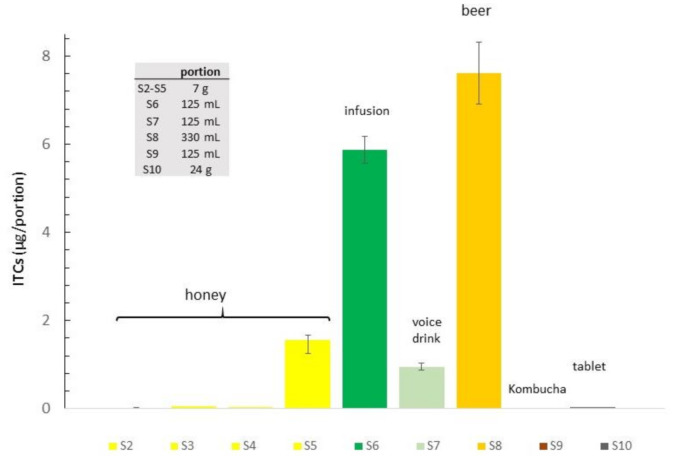
Comparison of released ITCs (isothiocyanates) content in single portions of food and drink preparations. Full description of samples S1–S10 is reported in Table 2.

The overall results of ITCs in food and drink preparations are summarised in Figure 3. 

## 4. Discussion

The rational design of functional food and drink requires an increasing knowledge about the molecular mechanism of action of specific active compounds and their biological targets. In this context, the research about taste and somatosensory active compounds is very active; in fact, several taste and somatosensory receptors are expressed ectopically and are therefore involved not only in the sensory profile of a food but also in post-ingestion metabolic and biochemical pathways. 

Somatosensory active compounds are gaining a relevant role in this area. The TRP ion channels are widely expressed sensors for temperature, somatosenses, sound, touch, and propriception [33]. The TRPA1 ion channel is responsible for the somatosensation of pungency of several spices and plants; it is involved, together with TRPVs, in mediating inflammatory and neurogenic pain [34,35].

*Sisymbrium officinale* contains glucosinolates and isothiocyanates, which are both characterised by a peculiar taste and some specific biological activity. The taste and flavor perceptions of glucosinolates, isothiocyanates, and related compounds have been recently reviewed [36]. Whereas it is generally accepted that many GSL compounds are bitter and ITCs impart pungency, this is not generally applicable to all compounds of these two families. Moreover, GSLs are phytochemical precursors to many compounds, not just ITCs; therefore, the overall sensory effects of a food preparation can hardly be correlated with a single product or family of products. 

The analysis of GLS in food has been extensively reviewed [37], whereas analysis of released ITCs is not so well standardised. Nevertheless, there is growing evidence that ITCs exert antioxidant, anti-inflammatory, and anticancer activities; therefore, the highest benefit of cruciferous foods occurs when they are consumed raw, avoiding the degradation of the enzyme myrosinase by cooking or processing [17].

Therefore, the idea to use SO as an ingredient for functional food and drink is based on the rationale of using the pungent volatile ITCs as active compounds to be released as “ready to use” volatile bioactives during assumption. The HS-SPME-GC/MS methodology allowed us to analyse the volatilome of several model food and drink enriched with SO in order to identify the active principles. Model food and drinks were chosen taking into account the possible practical use, the overall taste profile, and the combination with other useful ingredients such as honey. 

All the plant material can be contaminated by different microorganisms, including pathogenic ones posing a risk to the health of the consumers. In this study, we analyse the hygienic quality of *Sisymbrium officinale* through different indicators. The low levels of total bacterial count (<10^4^ CFU/g) are indicative of an acceptable quality [26]. The results obtained from yeast and moulds, *E. coli* and *B. cereus* counts confirmed that all the plant materials can be used as food ingredient, since all the values are below the required limits to consider these kinds of products as safe.

The overall results show that fresh seeds and flowers can be used to prepare formulates having similar or higher ITCSs content compared to that released by commercial tablets. Fermentation can remove the ITCS as in the case of kombucha or not, as in the case of beer. Some preliminary information on the role of temperature and grinding, particularly in the case of seeds, was obtained. 

These results can contribute to strengthen the relationship between chemosensory sciences, medicine, and food technology and to design new applications of somatosensory ingredients in the preparation of targeted functional food. 

## Figures and Tables

**Table 1:** Effects of meshing, temperature and time on release of isothiocyanates (ITCs) from fresh flowers of SO (S1) in water.

Sample	Grinding (Yes/No)	T (°C)	Time (min)	Total ITCs (µg/g)
S1/1	no	37	10	0.002
S1/2	yes	37	10	0.804
S1/3	yes	80	10	0.126
S1/4	no	80	5	0.140
S1/5	no	80	10	0.150

Total ITCs are reported as µg/g of fresh flowers. All measurements were made in triplicate. Standard deviation ≤10% in all samples.

**Table 2:** Analysis of IPITC (2) and SBITC (4) released in food and drink preparations. Total ITCs (2 + 4) are reported as µg/Kg of preparation.

Entry	Samples ^1^	2 (µg/kg)	4 (µg/kg)	Total ITCs (2 + 4) (µg/kg)	Portion	Total ITCs (2 + 4) (µg/Portion)
S2	Honey FW	3.10 ± 0.63	0.25 ± 0.01	3.51	7 g	0.02
S3	Honey FG	6.11 ± 0.65	0.70 ± 0.09	6.71	7 g	0.05
S4	Honey SW	3.98 ± 1.13	0.30 ± 0.18	4.01	7 g	0.03
S5	Honey SG	204.06 ± 17.90	18.28 ± 3.50	222.34	7 g	1.55
S6	Infusion ^2^	38.56 ± 8.51	8.29 ± 1.00	46.84	125 mL	5.85
S7	Voice drink	4.55 ± 1.13	3.06 ± 1.60	7.61	125 mL	0.95
S8	Beer	19.64 ± 0.44	3.46 ± 0.76	23.11	330 mL	7.62
S9	Kombucha	nd	nd	-	-	-
S10	Tablet ^3^	1.71 ± 0.11	0.20 ± 0.02	1.91	1 (24 g)	0.04

^1^ Legenda: F = flowers; S = seeds; W = whole; G = grinded. ^2^ Infusion made with commercial dry herb. ^3^ Commercial tablet. All measurements were made in triplicate; average values are reported. nd = not detectable; - = not applicable.

**Table 3:** Microbial counts of SO seeds, leaves, and flowers.

	Leaves	Flowers	Seeds	Reference
Total bacterial count	4.1 × 10^3^ CFU/g	3.4 × 10^3^ CFU/g	2.5 × 10^3^ CFU/g	[28]
Yeasts and moulds	<10^2^ CFU/g	<10^2^ CFU/g	<10^2^ CFU/g	[28]
*Escherichia coli*	<10 CFU/g	<10 CFU/g	<10 CFU/g	[29]
*Bacillus cereus*	<10^2^ CFU/g	<10^2^ CFU/g	<10^2^ CFU/g	

## Data Availability

The data presented in this study are available in this article and supplementary material.

## Supplementary Materials

The following are available online at https://www.mdpi.com/2304-8158/10/2/308/s1, Figure S1: calibration curves for ITCS: (a) concentration range: 0.25–1 ppm; (b) concentration range: 5–40 ppm; Figure S2: operative conditions for GCMS; Figure S3: Operative conditions for autosampling; Figure S4: mass spectrum of IPITC; Figure S5 Identification (a) and spectra (b) of unknown peaks; Table S1: additional data for Table 1.
