# Supplementary material for: Sisymbrium Officinale (the Singers’ Plant) as an Ingredient: Analysis of Somatosensory Active Volatile Isothiocyanates in Model Food and Drinks
Source: Foods. 2021 Feb 3;10(2):308. doi: 10.3390/foods10020308 (PMC7913150; doi:10.3390/foods10020308)
Supplement: Supplementary file 1 [file foods-10-00308-s001.pdf]

SUPPLEMENTARY MATERIAL

Figure S1. Calibration curves for ITCS. (**a**) concentration range: 0.25-1 ppm; (**b**) concentration range: 5-40 ppm.

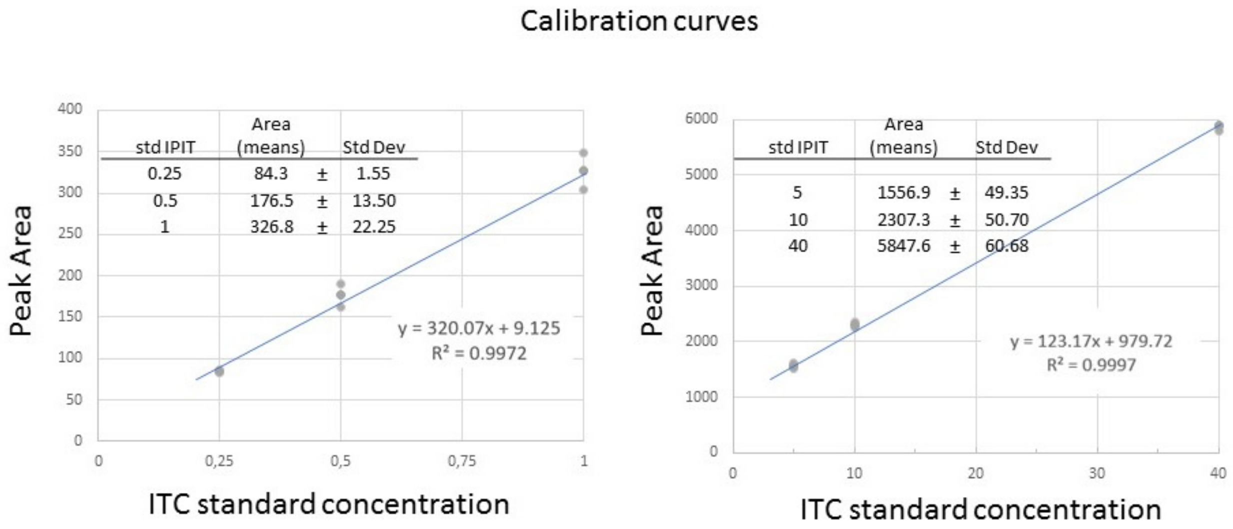

(a) (b)

Figure S2: operative conditions for GCMS

Oven	
Equilibration Time	0.25 min
Oven Program	On
40 °C for 2 min	
then 15 °C/min to 90 °C for 0 min	
then 15 °C/min to 170 °C for 0 min	
then 15 °C/min to 240 °C for 4 min	
Run Time	19.333 min

No Injectors

Front SS Inlet He		
Mode	Split	
Heater	On	200 °C
Pressure	On	7.7626 psi
Total Flow	On	9 mL/min
Septum Purge Flow	On	3 mL/min
Gas Saver	On	15 mL/min After 6 min
Split Ratio	5 :1	
Split Flow	5 mL/min	

Thermal Aux 2 {MSD Transfer Line}	
Heater	Off
Temperature Program	Off
230 °C for 0 min	
Run Time	19.333 min

Figura

Column #1
DB-5: 1479.69599
DB-5
325 °C: 30 m x 250 µm x 0.25 µm
In: Front SS Inlet He
Out: Vacuum

(Initial)	40 °C
Pressure	7.7626 psi
Flow	1 mL/min
Average Velocity	35.143 cm/sec
Holdup Time	1.4227 min
Flow Program	On
1 mL/min for 0 min	
Run Time	19.333 min

Figura 2.5: Condizioni gascromatografiche impiegate

Solvent Delay	: 0.50 min		
EMV Mode	: Relative		
Relative Voltage	: 0		
Resulting EM Voltage	: 1812		
[Scan Parameters]			
Low Mass	: 40.0		
High Mass	: 300.0		
Threshold	: 150		
Sample #	: 2	A/D Samples	4
[MSZones]			
MS Source	: 250 C	maximum 250 C	
MS Quad	: 150 C	maximum 200 C	

Figura 2.6: Parametri di acquisizione ms

Figure S3: Operative conditions for autosampling

```
                      CYCLE DETAILS
                     ---------------
        Pre Incubation Time (s): 60
         Incubation Temp. (°C): 80
   Pre Inc Agitator Speed (rpm): 250
           Agitator On Time (s): 10
          Agitator Off Time (s): 10
     Vial Penetration in mm (µl): 22
            Extraction Time (s): 300
                      Desorb to: GC Inj1
Injection Penetration in mm (µl): 54
            Desorption Time (s): 150
                 GC Runtime (s): 300
```

Figura 2.7: Impostazioni di microestrazione utilizzate per i campioni infusi

```
                      CYCLE DETAILS
                     ---------------
        Pre Incubation Time (s): 60
         Incubation Temp. (°C): 37
   Pre Inc Agitator Speed (rpm): 250
           Agitator On Time (s): 10
          Agitator Off Time (s): 10
     Vial Penetration in mm (µl): 22
            Extraction Time (s): 600
                      Desorb to: GC Inj1
Injection Penetration in mm (µl): 54
            Desorption Time (s): 180
                 GC Runtime (s): 300
```

Figura 2.8: Impostazioni di microestrazione utilizzate per i campioni non infusi

Figure S4: Spectrum of IPITC

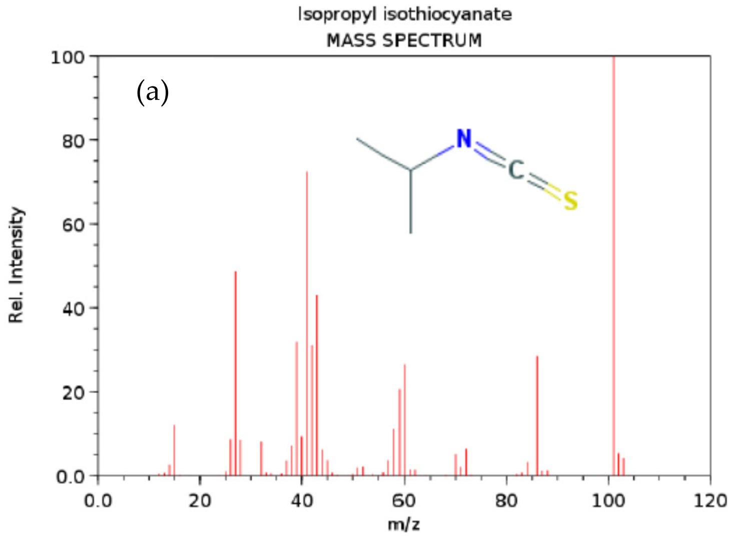

Figura 3.6: Spettro di massa di Isopropil-Isotiocianato

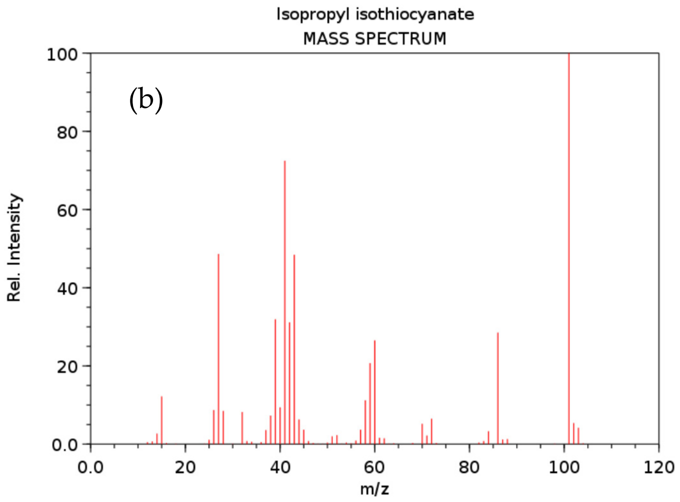

NIST Chemistry WebBook (https://webbook.nist.gov/chemistry)

Comparison, Mass spectrum of iPITC (a) from NIST (b)

Figure S5: Identification (a) and spectra (b) of unknown peaks

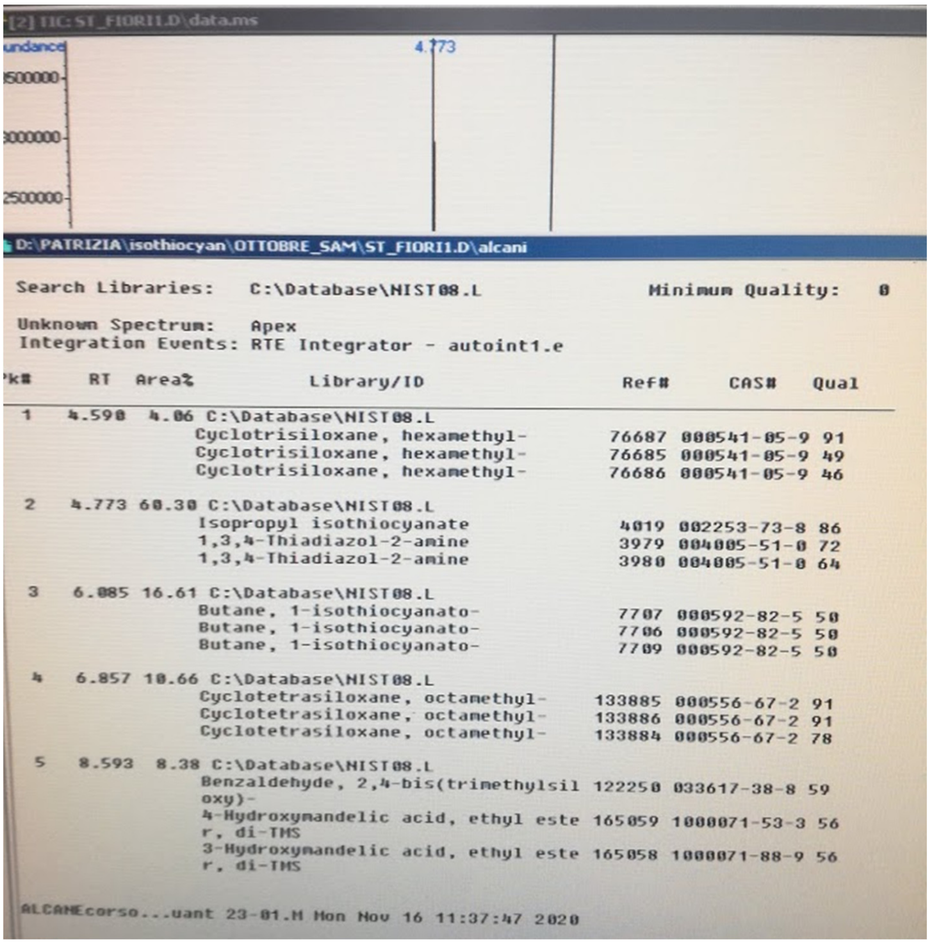

(a)

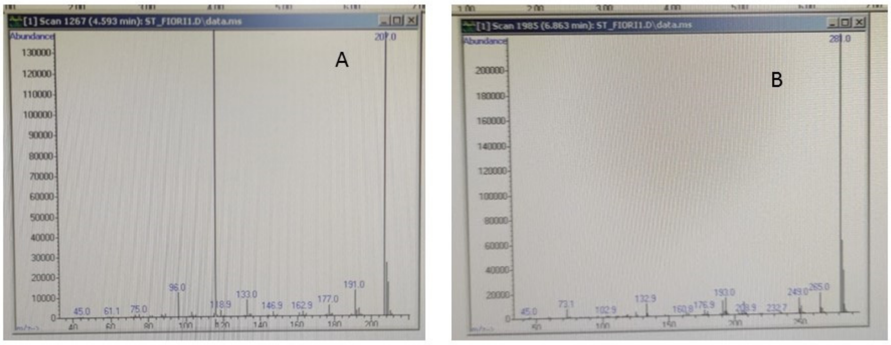

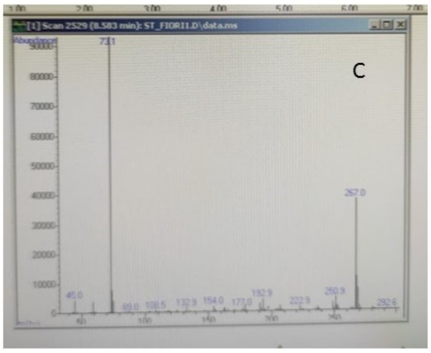

GCMS spectra of peaks at 4.59, 6.86 and 8.58 min of TIC shown in **Figure 2** correspond to some impurity (silyl derivatives compounds)

(b)

Table S1: additional data to Table 1

**sample**	**Total ITCs (μg/g)**
S1/1	0.002 ± 0.00018
S1/2	0.804 ± 0.07
S1/3	0.126 ± 0.01
S1/4	0.140 ± 0.09
S1/5	0.150 ± 0.01

Data are the means of 3 replicates ± SD
